# Supplementary material for: From sequence to enzyme mechanism using multi-label machine learning
Source: BMC Bioinformatics. 2014 May 19;15:150. doi: 10.1186/1471-2105-15-150 (PMC4229970; doi:10.1186/1471-2105-15-150)
Supplement: Additional file 2 — Java code of ml2db. Additional file ml2db_code.tar.gz contains the Java source code to run the multi-label machine learning experiments and save the results to database. The code’s Javadoc is included. [file 1471-2105-15-150-S2.zip › additional file 2/ml2db/ml2db/doc/main/ml2db/mulan/mulanarff/class-use/MulanDataSetDbLoader.html]

Uses of Class main.ml2db.mulan.mulanarff.MulanDataSetDbLoader


JavaScript is disabled on your browser.


- Overview
- Package
- Class
- Use
- Tree
- Deprecated
- Index
- Help

- Prev
- Next

- Frames
- No Frames

- All Classes

## Uses of Class main.ml2db.mulan.mulanarff.MulanDataSetDbLoader

- Packages that use MulanDataSetDbLoader

  | Package | Description |
  | --- | --- |
  |  |  |
  | --- | --- |
  | main.ml2db.mulan.mulanarff |  |
  | test.ml2db.mulan.mulanarff |  |
- - ### Uses of MulanDataSetDbLoader in main.ml2db.mulan.mulanarff

    Methods in main.ml2db.mulan.mulanarff that return MulanDataSetDbLoader

    | Modifier and Type | Method and Description |
    | --- | --- |
    |  |  |
    | --- | --- |
    | `MulanDataSetDbLoader` | MulanDataSetManager.`getDataLoader()` |
  - ### Uses of MulanDataSetDbLoader in test.ml2db.mulan.mulanarff

    Methods in test.ml2db.mulan.mulanarff that return MulanDataSetDbLoader

    | Modifier and Type | Method and Description |
    | --- | --- |
    |  |  |
    | --- | --- |
    | `static MulanDataSetDbLoader` | MulanDataSetDbLoaderTest.`getDbLoader()` |

- Overview
- Package
- Class
- Use
- Tree
- Deprecated
- Index
- Help

- Prev
- Next

- Frames
- No Frames

- All Classes
